# Evaluation of a training intervention to improve cancer care in Zimbabwe: Strategies to Improve Kaposi Sarcoma Outcomes (SIKO), a prospective community‐based stepped‐wedge cluster randomized trial

**DOI:** 10.1002/jia2.25998

**Published:** 2022-08-26

**Authors:** Katherine R. Sabourin, Margaret Borok, Samantha Mawhinney, Maxwell Matimba, Francis Jaji, Suzanne Fiorillo, Dickson D. Chifamba, Claudios Muserere, Busisiwe Mashiri, Chenjerai Bhodheni, Patricia Gambiza, Rachael Mandidewa, Mercia Mutimuri, Ivy Gudza, Matthew Mulvahill, Camille M. Moore, Jean S. Kutner, Eric A. F. Simões, Thomas B. Campbell

**Affiliations:** ^1^ University of Colorado School of Medicine Aurora Colorado USA; ^2^ Faculty of Medicine and Health Sciences University of Zimbabwe Harare Zimbabwe; ^3^ University of Zimbabwe Clinical Research Centre Harare Zimbabwe; ^4^ Island Hospice and Healthcare Harare Zimbabwe; ^5^ National Jewish Health Denver Colorado USA; ^6^ Colorado School of Public Health Aurora Colorado USA

**Keywords:** Kaposi sarcoma, training intervention tools, palliative care, primary community care, KS, HIV

## Abstract

**Introduction:**

Most Zimbabweans access medical care through tiered health systems. In 2013, HIV care was decentralized to primary care clinics; while oncology care remained centralized. Most persons in Zimbabwe with Kaposi sarcoma (KS) are diagnosed late in their disease, and the prognosis is poor. Little is known about whether educational interventions could improve KS outcomes in these settings.

**Methods:**

Interventions to improve KS detection and management were evaluated at eight Zimbabwe primary care sites (four rural/four urban) that provided HIV care. Interventions included a standardized KS clinical evaluation tool, palliative care integration, standardized treatment and improved consultative services. Interventions were implemented between February 2013 and January 2016 using a randomized stepped‐wedge cluster design. Sites were monitored for KS diagnosis rates and KS outcomes, including early diagnosis (T0 vs. T1 tumour stage), participant retention and mortality. Analyses controlled for within‐clinic correlations.

**Results:**

A total of 1102 persons with suspected KS (96% HIV positive) were enrolled: 47% incident (new diagnosis), 20% prevalent (previous diagnosis) and 33% determined as not KS. Early (T0) diagnosis increased post‐intervention, though not significant statistically (adjusted odds ratio [aOR] = 1.48 [95% confidence interval (95% CI): 0.66–3.79], *p* = 0.37). New KS diagnosis rates increased 103% (95% CI: 11–273%), *p* = 0.02) post‐intervention; although paired with an increased odds of incorrectly diagnosing KS (aOR = 2.08 [95% CI: 0.33–3.24], *p* = 0.001). Post‐intervention, non‐significant decreases in 90‐day return rates (adjusted hazard ratio [aHR] = 0.69 [95% CI: 0.38–1.45], *p* = 0.21) and survival (aHR = 1.36 [95% CI: 0.85–2.20], *p* = 0.20) were estimated.

**Conclusions:**

KS training interventions at urban and rural Zimbabwe decentralized primary care clinics significantly increased overall and incorrect KS diagnosis rates, but not early KS diagnosis rates, 90‐day return rates or survival.

## 1 INTRODUCTION

Despite the rollout of antiretroviral therapy (ART) in 2004, AIDS‐associated Kaposi sarcoma (AIDS‐KS) remains a major cause of morbidity and mortality in Zimbabwe [1, 2]. Late presentation of disease, concomitant opportunistic infections, and limitations in ART and chemotherapy access lead to unique challenges and high mortality rates for AIDS‐KS in Africa [3, 4, 5, 6, 7, 8, 9]. Reducing the AIDS‐KS burden in Zimbabwe and other African countries requires evidence‐based recommendations and strategies that address the prevention, screening, early diagnosis and treatment of this malignancy, particularly in primary care settings.

In Zimbabwe, the population is mostly rural and obtains medical care, including human immunodeficiency virus (HIV) diagnosis and ART, through a tiered system of local health centres, district, provincial and central hospitals in which nurses and community health workers are the initial primary healthcare contacts [10]. Zimbabwe's HIV Treatment and Care Programme expanded rapidly from its rollout in 2004, in line with the national policy of provision of services at the primary healthcare level. The National HIV Strategic Plan (2013–2017) details extension of access to testing and treatment at this level, training of healthcare providers and integration of primary healthcare services for HIV in the tiered system of referral for centralized, tertiary care.

The National ART Guidelines were amended in 2016 to reflect the Ministry of Health and Child Care commitment to the Global Fast Track 90‐90‐90 targets by 2020 and for consistency with World Health Organization recommendations that all HIV‐positive persons receive ART. Although nurses overseeing ART are trained on the rollout, indications, effects and toxicities of ART drugs, there is little training on the identification, diagnosis and management of HIV‐related malignancies. Training of healthcare workers in cancer detection, treatment and end‐of‐life care is required to reduce the burden of disease and improve outcomes and survival in low‐ and middle‐income countries [11].

To improve outcomes of AIDS‐KS in Zimbabwe and other African settings, KS screening, diagnosis and treatment recommendations must be integrated into primary HIV care settings. Strategies are needed to increase early‐stage KS diagnosis, when it is most likely to respond to ART alone and thereby reduce the need for expensive, difficult to access and potentially toxic chemotherapy. These strategies must be feasible, simple and sustainable in resource‐constrained primary care settings with high HIV burdens. We developed a package of training interventions focused on KS recognition and improved symptom control targeted at primary HIV care providers. We conducted a community‐based randomized stepped‐wedge cluster trial to ensure the intervention was received by all involved clinics. Using this format, we aimed to identify whether training healthcare workers in AIDS‐KS diagnosis and care improved the health outcomes of people with AIDS‐KS.

## 2 METHODS

### 2.1 Study oversight and monitoring

Study design details are available at ClinicalTrials.gov NCT01764360. The study was approved by the institutional review boards of the Medical Research Council of Zimbabwe, University of Colorado Anschutz Medical Campus and the Joint Parirenyatwa Hospital and University of Zimbabwe College of Health Sciences. Written, informed consent was obtained from all study participants.

### 2.2 Interventions

A package of interventions was developed to improve KS care by providing training in clinical recognition and evaluation of KS, palliative care and symptom control (see File S1). The Intervention Package comprised three components: (1) A KS Standardized Evaluation (KS‐SE) [12]. Early KS diagnosis is highly dependent on clinical detection of suspected KS lesions. The KS‐SE was developed with input from Zimbabwean clinicians experienced in KS care to improve bedside diagnostic skills to assess common clinical presentations of KS. The KS‐SE promoted a quick, comprehensive physical examination of patients with AIDS‐KS, and was recommended for use at diagnosis and annually thereafter to document treatment response. (2) Palliative care education and integration into the primary care of persons with AIDS‐KS. The training was modified to focus on AIDS‐KS symptomatology and delivered by specially trained and experienced Zimbabwean doctors and nurses whose main practice is in the provision of home‐based palliative care. (3) Consultative and educational services for primary care providers in rural areas. Training modules incorporating KS recognition, particularly early KS diagnosis, treatment, symptom control and palliative care, were developed by a team of Zimbabwean nurses and doctors experienced in KS care. In addition, each site was encouraged to consult with the study team for assistance in the diagnosis and management of AIDS‐KS.

### 2.3 Trial design

**Figure 1:**
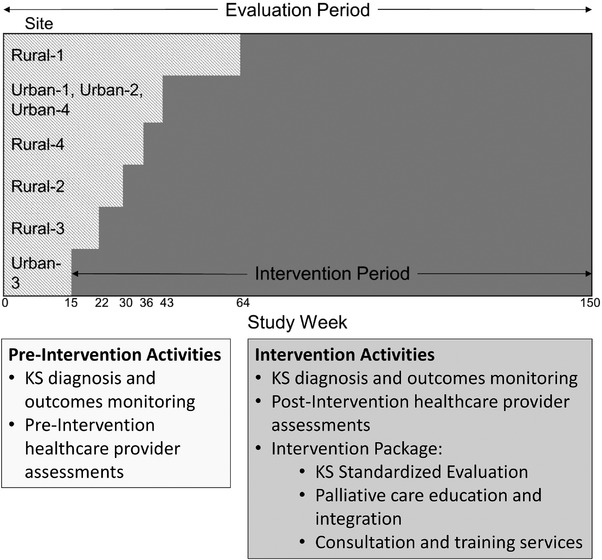
Timeline for study implementation and completion for randomized stepped‐wedge cluster trial. Each site was monitored for Kaposi sarcoma diagnoses and outcomes throughout the entire study period (evaluation period, weeks 0–150). The light‐shaded area shows the time when each of the eight sites was monitored prior to implementation of the Intervention Package (pre‐intervention period). The dark‐shaded area shows the time when sites were monitored after the implementation of the Intervention Package (intervention period). The time of intervention implementation was randomly assigned for each site; because the Urban‐1, Urban‐2 and Urban‐4 sites are in close geographic proximity, and share staff and patients, these three sites were randomized as a cluster. The Urban‐3 site was the first site to begin the intervention at week 15. The last site to begin the intervention was the Rural‐1 site at week 64.

The Intervention Package impact on AIDS‐KS outcomes was evaluated by a stepped‐wedge randomized cluster trial design (Figure 1) [13, 14]. Monitoring of KS diagnoses and outcomes began in February 2013, 15 weeks before the first implementation of the Intervention Package, and continued until the studies end in January 2016 (evaluation period). The evaluation period was 150 weeks for all sites. Sites were randomized to begin the Intervention Package at different times such that the intervention was eventually implemented at all sites during the study. The time from initial monitoring to intervention initiation ranged from 15 to 64 weeks (pre‐intervention period). The time from the start of the intervention to the end of the study ranged from 86 to 135 weeks (intervention period).

### 2.4 Study settings

The Intervention Package was implemented at eight primary care sites (four rural and four urban). Urban sites were located within the metropolitan area of Harare and rural sites within a 200 km radius of Harare. All sites received support for HIV care through the President's Emergency Plan for AIDS Relief (PEPFAR) [15], provided primary HIV care services, including HIV staging and assessment, initiation and follow‐up of ART, and had a minimum census of 1000 persons living with HIV receiving care.

### 2.5 Randomization

The intervention order for urban and rural sites was block randomized. Within blocks, sites were then randomized to obtain the order for implementation of the Intervention Package. This design ensured that urban and rural sites had a similar number of weeks in the pre‐intervention and intervention periods. Three of the four urban sites were grouped together in the randomization as they were in close geographic proximity and shared staff and patients.

### 2.6 Participants

Persons of all ages with suspected or confirmed KS who received care at the participating sites at any time during the study period were asked to participate.

### 2.7 Study procedures

The study included a Monitoring Team and Intervention Team. Team membership was mutually exclusive and worked independently. The Monitoring Team visited each site weekly throughout the evaluation period. Monitors were blinded to the site randomization results and to when each site began the intervention period. At site visits, the monitors distributed study forms and trained site staff on form completion and keeping daily logs of the total number of patient visits, new KS diagnoses (incident cases) and patients with previously known KS (prevalent cases). They did not provide training in any aspect of KS diagnosis or treatment.

The Intervention Team included at least one doctor experienced in palliative and AIDS‐KS patient care and one nurse. At each site, the Intervention Team conducted trainings directed at all levels of primary care providers (doctors, nurses, allied health professionals and ancillary staff). Training methods were interactive, case‐based and included photographs of common presentations of KS, and discussion of KS cases with various HIV‐ and KS‐related complications. Trainings emphasized early recognition of the disease, including demonstration of a focused full‐body examination for KS, potential KS mimics, and appropriate management of KS and complications including concomitant opportunistic infections. Healthcare providers were watched and given feedback as they did a KS‐focused clinical examination after a training demonstration. In the first month of the intervention period for each site, training included three 4‐hour sessions; thereafter, training was done monthly for 2–4 hours per session through the end of the study. The Intervention Team also provided training to community health workers and traditional healers on KS recognition and referral for medical treatment and distributed posters and brochures in the community to increase awareness of KS.

Prior to the start of training and at subsequent follow‐up trainings, healthcare providers were asked to self‐rate their knowledge and skills in six areas (performance of a KS‐specific history and examination, clinical detection of KS, assessment of KS symptoms, performance of KS staging, management of KS symptoms and principles of palliative care) using a 5‐point Likert scale (ranging from 1—“I have no knowledge or skill” to 5—“I am an expert”).

To confirm KS diagnosis, doctors and nurses were trained to do punch biopsies of suspected KS skin lesions, and sites were provided with the necessary supplies for specimen collection, storage and transport to pathology at the University of Zimbabwe College of Health Sciences. Biopsies were read by a histopathologist who had completed the AIDS Clinical Trials Group/AIDS Malignancy Consortium training in KS histopathology [16]. Sites were encouraged to obtain biopsies from at least one skin lesion for all KS suspects. When biopsies were not obtained, KS diagnosis was made by the treating clinician and confirmed by expert opinion after a clinical examination by a study team physician experienced in the evaluation and treatment of KS. Confirmed KS cases, whether a biopsy or clinically confirmed, were classified as prevalent if diagnosed prior to the start of the study and incident if newly diagnosed after study initiation.

### 2.8 Outcomes

We hypothesized that the training interventions would lead to more active KS surveillance by primary care providers resulting in increased overall and early‐stage KS diagnoses. Analyses included confirmed AIDS‐KS only. Outcomes measured were the proportion of confirmed incident (newly diagnosed) KS over time and the proportion of confirmed incident KS identified at stage T0 (T0/(T0 + T1)) with T0 and T1 defined by ACTG criteria [17]. We also hypothesized that the intervention would improve AIDS‐KS survival and retention in care among incident KS cases. Retention was measured as the time to the second visit, usually a month from the diagnostic visit, which was required for patient review and administration of chemotherapy when needed, with censoring at the maximum follow‐up time. The number of required clinic visits varied according to individual clinical need, with not often more than four chemotherapy pulses given monthly, reverting to visits every 3 months depending on clinical disease and treatment response. Non‐death loss‐to‐care was included because loss‐to‐care in ART programmes in African settings is associated with a high risk of mortality [18]. Death among participants with incident confirmed KS was evaluated separately.

### 2.9 Statistical methods

Non‐linear mixed effects models were used to account for the site, subject and time effects [14]. Time to event outcomes during the evaluation and intervention periods utilized Cox frailty models, accounting for site effects and time‐varying covariates, including transitions from evaluation to intervention periods. Step functions were used to account for non‐proportional hazards. Given incomplete participant and site ID and a lower response rate post‐intervention, a Welch's two‐sample *t*‐test was used to compare self‐ratings of KS knowledge pre‐ and post‐intervention.

## 3 RESULTS

### 3.1 Intervention delivery and uptake

The Intervention Package was implemented at each site as shown in Figure 1. Across eight sites, 2534 healthcare providers attended at least one training session, including 1658 nurses, 454 primary healthcare workers, 280 paramedics and 142 medical doctors. In addition, 205 traditional medicine practitioners and 47 community advocates received training. Prior to the first training session, 771 KS knowledge assessment surveys were completed by healthcare providers with 170 surveys completed at subsequent sessions (Table 1). At baseline, most healthcare providers indicated no or little knowledge or skill in each of the six areas (Likert scale: 1.4–2.0). At subsequent assessments, most participants reported at least some knowledge in each area (Likert scale: 2.6–3.1).

**Table 1:** Healthcare provider self‐rating of Kaposi sarcoma knowledge

	Pre‐intervention (*n* = 771)^a^, ^,b^	Post‐intervention (*n* = 170)^b^, ^,c^	*p*‐value
How would you rate your knowledge and skills in the following areas?^d^			
Principles and practice of palliative care	2.0 (0.9)	2.7 (1.1)	<0.001*
Performance of a KS‐specific history and exam	1.6 (0.8)	2.8 (1.1)	<0.001*
Assessment of KS symptoms	1.8 (0.8)	3.1 (1.1)	<0.001*
Detection of KS by history and exam	1.8 (0.8)	3.0 (1.1)	<0.001*
Performance of clinical staging of KS diseases	1.4 (0.7)	2.6 (1.0)	<0.001*
Management of KS symptoms	1.6 (0.8)	2.8 (1.1)	<0.001*

^a^ Pre‐intervention self‐assessments were performed on the first day of the training intervention but prior to any trainings.

^b^ Mean (standard deviation).

^c^ Post‐intervention self‐assessments were performed at 4, 24 or 48 weeks after implementation of the training intervention.

^d^ Site staff was asked to rate themselves as: I have no knowledge or skill (1); I have little knowledge or skill (2); I have some knowledge or skill (3); I have a lot of knowledge or skill (4); I am an expert (5).

* *p*<0.05 considered statistically significant.

### 3.2 Intervention effect on KS identification

**Figure 2:**
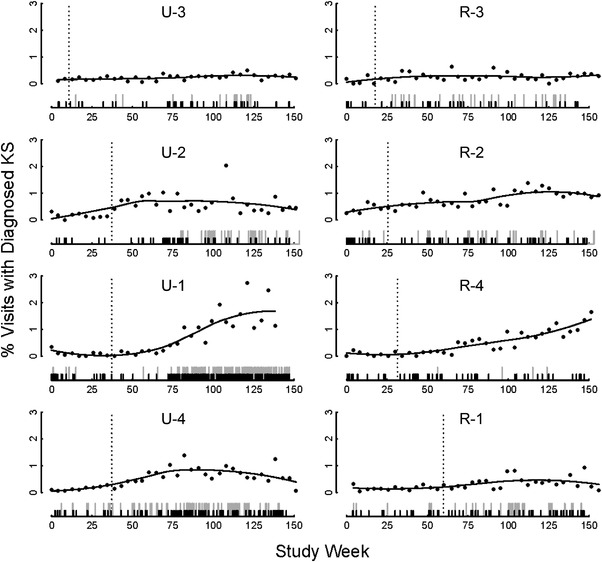
Effect of the training intervention on Kaposi sarcoma (KS) diagnosis rate. The proportion of all weekly HIV clinic visits that were patients with a suspected KS diagnosis is shown for the duration of the study period for each site. Vertical dashed lines indicate the time that the training intervention was introduced. Sites are grouped by urban (left panel) and rural locations (right panel). The tick mark rug indicates study enrolment times for confirmed KS cases (black tick marks) and participants initially identified as having KS but later determined to not have KS by expert opinion (grey tick marks).

**Figure 3:**
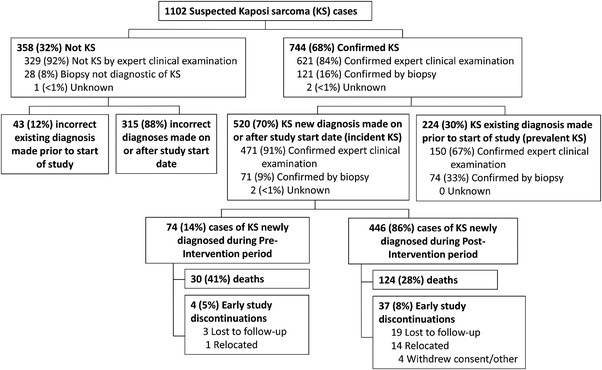
Diagram for identification of Kaposi sarcoma (KS) cases. A total of 1102 suspected cases of KS were evaluated during the combined pre‐intervention and intervention periods: 358 were determined to not be KS by expert opinion. Of the 744 confirmed KS cases, in 224 cases, the diagnosis of KS was made prior to study week 0. Of the 520 confirmed KS cases diagnosed after week 0, 74 were diagnosed during the pre‐intervention period and 446 during the intervention period.

Across the eight sites, 1102 individuals with suspected KS were identified. The proportion of HIV clinic visits with suspected KS increased during the intervention period at all sites although the size varied by site (Figure 2). KS diagnosis was confirmed by expert clinical examination or biopsy in 744 (68%) of 1102 KS suspects (Figure 3), including 520 (62%) of 834 new diagnoses (incident cases) and 224 (84%) of 267 existing diagnoses established prior to the study (prevalent cases). The time between provisional diagnosis and expert opinion or histopathology was 1–2 weeks (maximum 4 weeks). The mean rate of confirmed incident KS across sites increased from 2.1 (95% confidence interval [95% CI]: 1.3, 3.3) per week pre‐intervention to 4.2 (95% CI: 2.9, 6.4) per week in the intervention period (*p* = 0.021), although the frequency of incorrectly diagnosing someone with KS also increased from 19.9% to 34.3% (adjusted odds ratio [aOR] = 2.08; 95% CI: 1.33, 3.24, *p* = 0.001).

Of the 520 individuals with confirmed incident KS, 74 were enrolled pre‐intervention and 446 during the intervention period (Table 2). Incident KS cases were similar in gender, age, race and HIV status regardless of enrolment period. During the intervention period, a smaller percentage of incident KS cases were enrolled at a rural location (31% vs. 50%), had prior ART use (58% vs. 69%) or had current tuberculosis treatment (14% vs. 19%) compared to the pre‐intervention period.

**Table 2:** Baseline characteristics of confirmed new Kaposi sarcoma diagnoses made during the study evaluation period

	Pre‐intervention^a^ (*n* = 74)	Intervention^b^ (*n* = 446)
Female	31 (41.9%)	175 (39.2%)
Age^c^	37.5 (33.0; 43.0)	37.0 (32.0; 43.0)
Black African	74 (100%)	446 (100%)
Rural location	37 (50%)	138 (30.9%)
Prior antiretroviral therapy	51 (68.9%)	352 (57.6%)
Current treatment for tuberculosis	14 (18.9%)	60 (13.5%)
HIV antibody positive	74 (100%)	446 (100%)
CD4^+^ cells (per mm^3^)^c^, ^d^	179 (50; 330)	184 (59; 346)
Oral KS present	30 (42.3%)	220 (49.8%)
KS stage T0^a^ ^,e^	7 (9.5%)	52 (11.7%)

Note: All numbers given are *n* (%) unless otherwise specified.

^a^ New KS diagnoses were made prior to the date that the training intervention was introduced at the site where the KS evaluation occurred.

^b^ New KS diagnoses were made on or after the date that the training intervention was introduced at the site where the KS evaluation occurred.

^c^ Median with interquartile range (IQR).

^d^ CD4^+^ cell count data were available for 55 participants enrolled in the pre‐intervention period and 321 participants enrolled in the intervention period.

^e^ ACTG criteria for KS staging.

### 3.3 Intervention effect on early KS diagnosis

**Figure 4:**
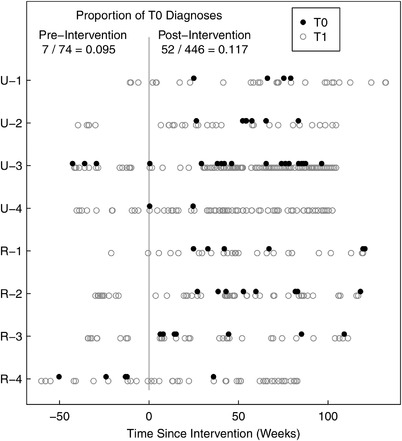
Kaposi sarcoma (KS) diagnoses relative to the time of implementation of the intervention for each clinic. Time of diagnosis is shown for the 520 new confirmed KS cases relative to the time of the intervention at each site (vertical shaded line). Filled circles are ACTG stage T0 KS; empty circles are ACTG stage T1. The proportion of T0 diagnosis in each period is shown.

The proportion of T0 stage KS increased from 9.5% pre‐intervention to 11.7% in the intervention period (Figure 4). In models adjusted for age, sex, clinic location (rural/urban), HIV status, time since HIV diagnosis and ART use, the odds of T0 stage confirmed KS diagnosis among incident cases, within a clinic, in the intervention versus pre‐intervention periods was 1.48 (95% CI: 0.63, 3.49; *p* = 0.37) (Table 3).

**Table 3:** Adjusted odds ratios for tumour stage at diagnosis (T0 vs. T1) for individuals with confirmed Kaposi sarcoma newly diagnosed at enrolment (*n* = 520)

	Odds ratio (95% confidence intervals)	*p*‐value
Enrolled during intervention period versus pre‐intervention period	1.48 (0.66, 3.79)	0.37
Age (per 10‐year increase)	0.91 (0.68, 1.21)	0.52
Female versus male sex	1.47 (0.83, 2.58)	0.18
Rural versus urban enrolment site	1.65 (0.69, 3.96)	0.18
Time since HIV diagnosis (per 1‐year increase)	1.05 (0.83, 1.18)	0.41
HIV negative versus HIV positive	0.38 (0.02, 2.03)	0.36
Prior antiretroviral therapy versus no prior antiretroviral therapy	0.69 (0.38, 1.26)	0.23

### 3.4 Intervention effect on retention in care and mortality

**Figure 5:**
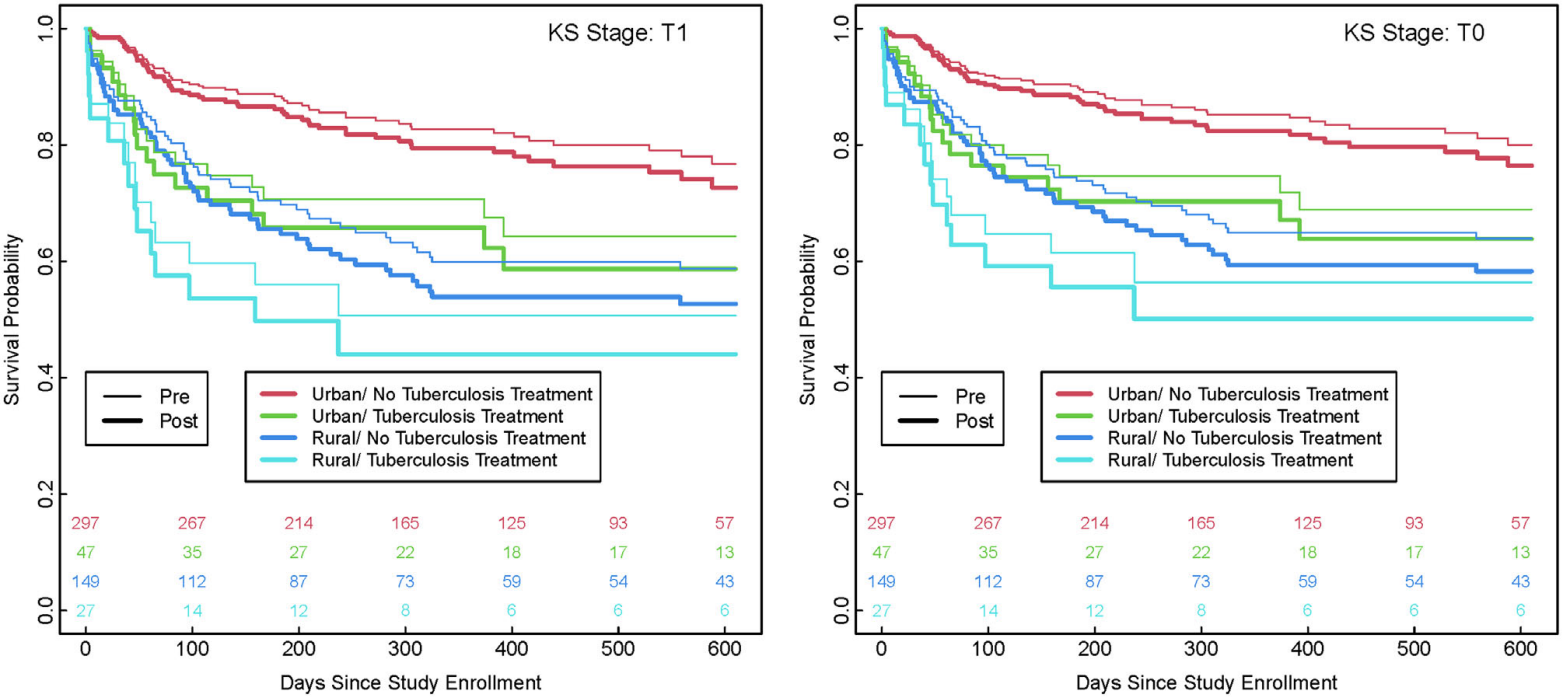
Adjusted Cox proportional hazards model of survival. Adjusted Cox proportional hazards model of survival of new (incident) Kaposi sarcoma (KS) cases by pre‐ and post‐intervention status (denoted by line width) with stratification by enrolment at a rural (vs. urban) clinic and tuberculosis status (denoted by colour). Adjustment covariates age, sex and time since HIV diagnosis were set to median values (age = 37 years, sex = male, time from HIV to KS = 0.92 years) with separate graphs for T0 and T1 status. Only the 520 confirmed incident (newly diagnosed) KS cases were included in the analysis. Censoring occurred at the maximum clinic visit.

Among confirmed incident KS cases, 5.4% had non‐death loss‐to‐care in the pre‐intervention period compared to 8.2% in the intervention period (Figure 3). However, the relative rate of return for a second clinic follow‐up increased over time for the intervention period compared to the pre‐intervention period: adjusted hazard ratio (aHR) = 0.67 (95% CI: 0.38, 1.21; *p* = 0.19) for the first 90 days; aHR = 1.37 (95% CI: 0.79, 2.39; *p* = 0.26) for 90–180 days; and aHR = 2.84 (95% CI: 1.47, 5.48; *p* = 0.002) after 180 days. Incident KS 1‐year mortality was 37% (95% CI: 32%, 42%) with 41% of deaths identified pre‐intervention compared to 28% in the intervention period. Among incident KS cases, the time to death aHR was 1.36 (95% CI: 0.85, 2.20; *p* = 0.20) adjusted for age, sex, T stage, time since HIV diagnosis and stratified by tuberculosis and clinic location to maintain proportional hazards (Figure 5).

## 4 DISCUSSION

To determine if healthcare worker training in AIDS‐KS diagnosis and care led to better health results for people with AIDS‐KS in Zimbabwe, we carried out one of the largest prospective trials of KS diagnosis and management in sub‐Saharan Africa to date. To improve knowledge across the healthcare spectrum, we trained nurses and doctors from HIV and non‐HIV‐focused clinics, pharmacists, rehabilitation technologists, administrative staff, community health workers and traditional healers. By conducting the intervention in both urban and rural clinics, our findings are generalizable to AIDS‐KS in Zimbabwe, where the majority of the population lives in rural areas. Healthcare providers self‐reported improved knowledge and ability to identify and manage KS post‐training. During the intervention period, diagnoses of KS increased and although earlier T0 stage new confirmed KS diagnoses also increased, this change was not statistically significant.

A small proportion of individuals newly diagnosed with KS in our study were identified at the T0 stage (11%), similar to prior studies in Africa [5, 6, 19, 20, 21], which may be indicative of barriers to accessing care rather than healthcare provider diagnostic skillsets. Distance to a clinic, prices of transportation or healthcare, lack of education and use of traditional healers [22, 23, 24] may lead to individuals waiting until late‐stage disease to seek out appropriate care. Traditional healers and community health workers were included in our educational programmes and we distributed posters and brochures to increase community awareness of KS but our intervention did not include other measures to decrease barriers to medical care access nor more active community engagement approaches. Outcomes in our study may have also been affected by scheduled rotations of clinical staff. Periodic rotation of new less experienced healthcare staff into the clinic while trained staff rotated out over the course of the study required continuous repetition of initial training modules and limited our ability to progress to more advanced training for many staff.

Although the intervention increased the overall rate of KS diagnosis, the rate of incorrectly diagnosing someone with KS also increased. Diagnosis of KS in African primary care settings is difficult as there are several mimicking disorders [25, 26], disseminated or pulmonary KS is hard to distinguish from tuberculosis [4, 27], and there is limited access to tumour biopsies for histologic diagnosis. Previous studies have reported up to 30% discordance between clinical and histopathologic KS diagnoses in South and East Africa [26, 28]. At our sites, the frequency of incorrect KS diagnosis was 16% prior to the study and 20% during the pre‐intervention period. The increased rate of incorrect diagnosis during the intervention period (35%) was unexpected since the intervention included training in KS clinical diagnosis after which healthcare providers reported feeling more confident in their abilities to diagnose KS, though the post‐intervention response rate was low (22%) which limits interpretation. However, the increase in overall KS diagnoses suggests that the intervention empowered primary care providers to examine a patient and attempt a diagnosis, even though not always KS. It is possible the response rate may have improved with further training, and that the programme may have needed to continue to have maximum effectiveness.

Despite skin biopsy training and the availability of punch needles and other biopsy supplies, only 16% of incident cases were confirmed by histopathology. We did not investigate the reasons for the low uptake of skin biopsies but suspect the cost of sending biopsies to a central pathology laboratory and the need for additional clinic visits to review biopsy results might have been inhibitory. In sub‐Saharan Africa, the positive predictive values of KS diagnosis by clinical examination compared to biopsy are estimated at 59–84%. Of individuals incorrectly diagnosed with KS requiring alternative treatment, up to 16–35% were considered clinically benign, and 5–6% had alternative diagnoses that were life‐threatening [28, 29, 30]. To improve the accuracy of KS diagnosis in Zimbabwe and other African settings, affordable, timely and scalable testing that is amenable for busy primary care clinics needs to be identified.

We did not detect statistically significant effects of the intervention on mortality. Overall mortality was high, potentially due to the large proportion of individuals presenting with advanced KS. Studies completed in sub‐Saharan Africa, including one in Zimbabwe, have reported increased mortality risk among individuals with more advanced tumour burden (T1) [5, 7, 9, 31]. Although study participants received ART, ART alone is not adequate treatment for advanced KS. Absent or inadequate chemotherapy has been associated with increased AIDS‐KS mortality [5, 6, 7, 8, 9, 32, 33]. Chemotherapy requires resources for travel to treatment facilities and payment for cancer therapy; unfortunately, these resources are not available to many AIDS‐KS patients in sub‐Saharan Africa [3]. The trend for increased time to death during the intervention, while not statistically significant, suggests that educational interventions targeted at African primary care providers have the potential to decrease mortality from KS. Programmes to reduce cancer morbidity and mortality in sub‐Saharan Africa should focus on continuing to build infrastructure and capacitating healthcare personnel to provide affordable, accessible and effective chemotherapy.

There was evidence that the intervention improved retention in care for newly diagnosed AIDS‐KS. The relative rate of return for a second clinic visit increased over time with incremental increases in the aHR measured at 3‐month intervals after starting the intervention, reaching statistical significance after 180 days. Trainings were provided multiple times at each site. Improvements in retention over time may reflect the time required for a sufficient number of site staff to participate in trainings and for the trained staff to garner experience in applying what they learned to their clinical practice. Repetition in training over time may lead to better improvements in knowledge and application of that knowledge in clinical practice.

This study had limitations. Since this study focused on determining the effects of healthcare provider training on the early diagnosis and subsequent health outcomes of individual patients, we collected individual participant but not provider data and so we were unable to identify groups of providers who were more accurate in their KS diagnoses. An incorrect KS diagnosis, either false negative or false positive, could have adversely affected clinical outcomes from lack of appropriate treatment or toxicities from unnecessary treatments. The high mortality does not suggest a high rate of misdiagnosis of KS for otherwise benign conditions. However, if participants had other life‐threatening conditions that were misdiagnosed as KS, incident mortality would have been overestimated. Although including both rural and urban clinics increased the generalizability of our results to AIDS‐KS in Africa, it is important to note that participants had to present to a participating site potentially excluding individuals with more severe KS or those unable to present to a clinic because of physical or monetary barriers, likely leading to an underestimate of T1 stage presentation and overall KS mortality. Our study was also insufficiently powered to detect modest effects on T0 diagnosis (OR<1·89) and on mortality and we cannot exclude the possibility that the intervention had effects smaller than what we identified. Although our study was conducted during the early decentralization of HIV treatment in Zimbabwe, initial KS diagnosis and treatment in Zimbabwe, and other African countries, remain largely decentralized and our findings remain relevant in the present day.

## 5 CONCLUSIONS

Training Zimbabwean healthcare providers in KS diagnosis and care increased confidence in their clinical abilities and improved retention in care. Basic knowledge regarding early KS diagnosis, the use of biopsy, treatments, such as chemotherapy, and symptom palliation should be included in standard curricula for primary healthcare providers in Zimbabwe and other African countries with a high burden of AIDS‐KS. Improvements in morbidity and mortality caused by AIDS‐KS in sub‐Saharan Africa will likely require a multi‐pronged approach that includes oncology training of health workers in rural areas, increased public knowledge of AIDS‐KS, and increased access to affordable cancer diagnostics and effective chemotherapies.

## COMPETING INTERESTS

No competing interests were reported by any authors.

## AUTHORS’ CONTRIBUTIONS

All authors made a considerable contribution to the study and manuscript. KRS contributed to data interpretation and manuscript writing and revision. MB and TBC were significantly involved with study design, data acquisition and interpretation, and manuscript writing and revision. CMM, JSK, EAFS and JH contributed substantially to study design, data acquisition and interpretation, and manuscript revision. SM supplied time and effort towards study design, data analysis and interpretation, and manuscript writing and revision. MM contributed significantly to data analysis and interpretation and manuscript revision. MM, FJ, SF, DDC, CM, BM, CB, PG, RM, MM and IG were substantially involved in data acquisition and interpretation, and manuscript revision. All authors have approved the final version of this manuscript to be published and agree to the accountability of all aspects of the work completed.

## FUNDING

Funding was provided by the National Cancer Institute, National Institutes of Health: grant 1R01CA172050. The funders had no role in study design, data collection or interpretation.

## DISCLAIMER

The findings of this study are solely the responsibility of the authors and do not necessarily represent the official views of NCI or NIH.

## Supporting information


**File S1**: SIKO KS Clinical Management Manual.

Information on file format. A PDF file containing the manual used during the described intervention for KS diagnosis and management.

## Data Availability

The data that support the findings of this study are available from the corresponding author upon reasonable request.
