# Supplementary material for: Evaluation of a training intervention to improve cancer care in Zimbabwe: Strategies to Improve Kaposi Sarcoma Outcomes (SIKO), a prospective community‐based stepped‐wedge cluster randomized trial
Source: J Int AIDS Soc. 2022 Aug 26;25(8):e25998. doi: 10.1002/jia2.25998 (PMC9418419; doi:10.1002/jia2.25998)
Supplement: Supplementary file 2 — Information on file format. A PDF file containing the manual used during the described intervention for KS diagnosis and management. [file JIA2-25-e25998-s001.pdf]

SIKO

KS Clinical

Management Manual

SIKO ZIMBABWE

KNOWLEDGE • DILIGENCE • INTEGRITY

SIKO
KS Clinical
Management Manual

Contents

1. Overview of KS in Zimbabwe
   1.1 Epidemiology
   1.2 Recognition
   1.3 Staging
   1.4 Treatment

2. KS Standardized Evaluation (KS-SE) and KS-SE Checklist

3. Palliative Care for KS

4. Biopsy of KS Lesions

5. KS Management Algorithm

6. Photography of KS Lesions

7. Obtaining KS and Palliative Specialty Consultation

1. KS in Zimbabwe

1.1 KS epidemiology in Zimbabwe

Kaposi's sarcoma (KS) is a type of cancer that is caused by infection with human herpesvirus 8 (HHV-8 or Kaposi's sarcoma-associated herpesvirus). HHV-8 infection is common in Zimbabwe but most people who are infected do not get KS. People who are infected with both HHV-8 and HIV are at high risk for developing KS. KS is one of the most common tumors reported to the Zimbabwe National Cancer Registry and is a major cause of mortality and morbidity in Zimbabwe. (see ZNCR Annual Reports)

1.2 KS recognition and clinical assessment

Clinical presentation of KS can include cutaneous (skin), oral, lymphatic or visceral organ disease, or combinations of disease at multiple sites. A detailed physical examination is essential for both detection of KS disease and assessment of the extent of tumor burden. Skin and mouth lesions are usually darkly pigmented, often reddish purple in color. The skin lesions may be flat (macules), raised (papules) or nodular. Although a KS diagnosis can often be made by clinical examination, KS lesions may be difficult to distinguish from other skin diseases (bacillary angiomatosis, lupus erythematosus) and a biopsy with histopathology is required for confirmation. Using a specific stain, it is possible to identify HHV-8 on the biopsy sample.
Photographs of common KS presentations are available in the next pages.

Please note that all photographs have been taken after permission has been obtained from the patient.

Common Sites for KS Lesions

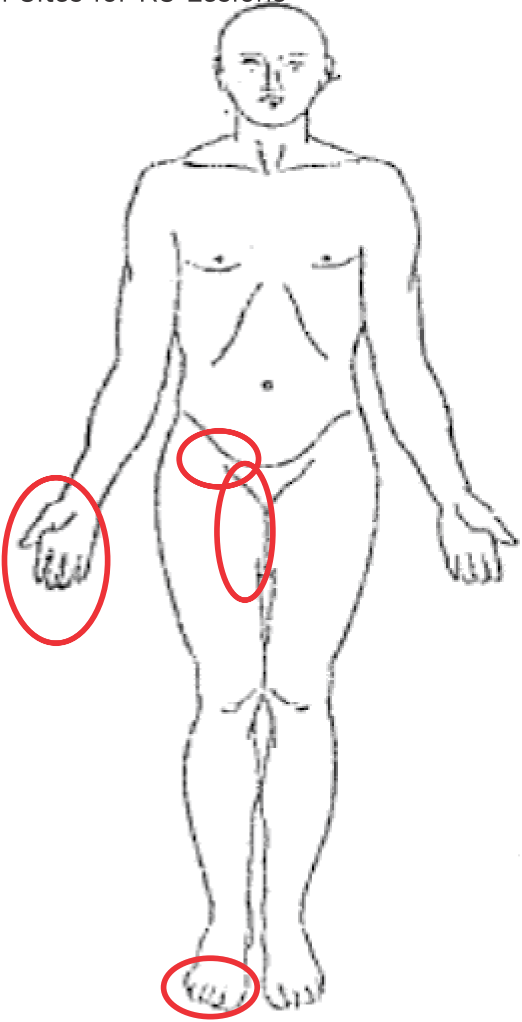

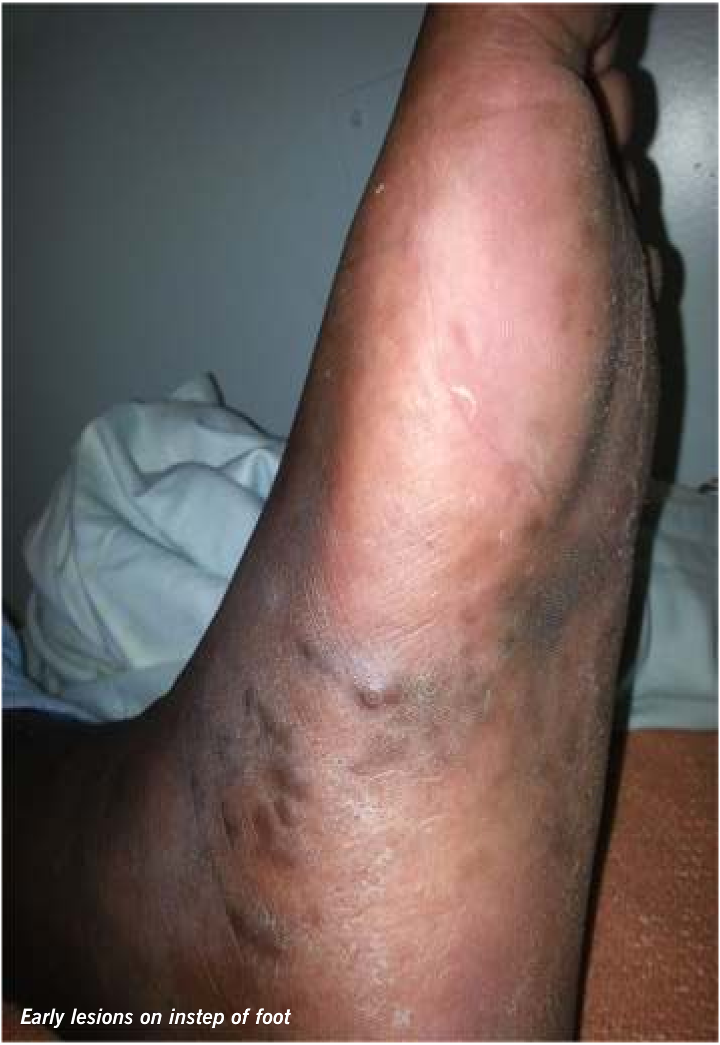

Early lesions on instep of foot

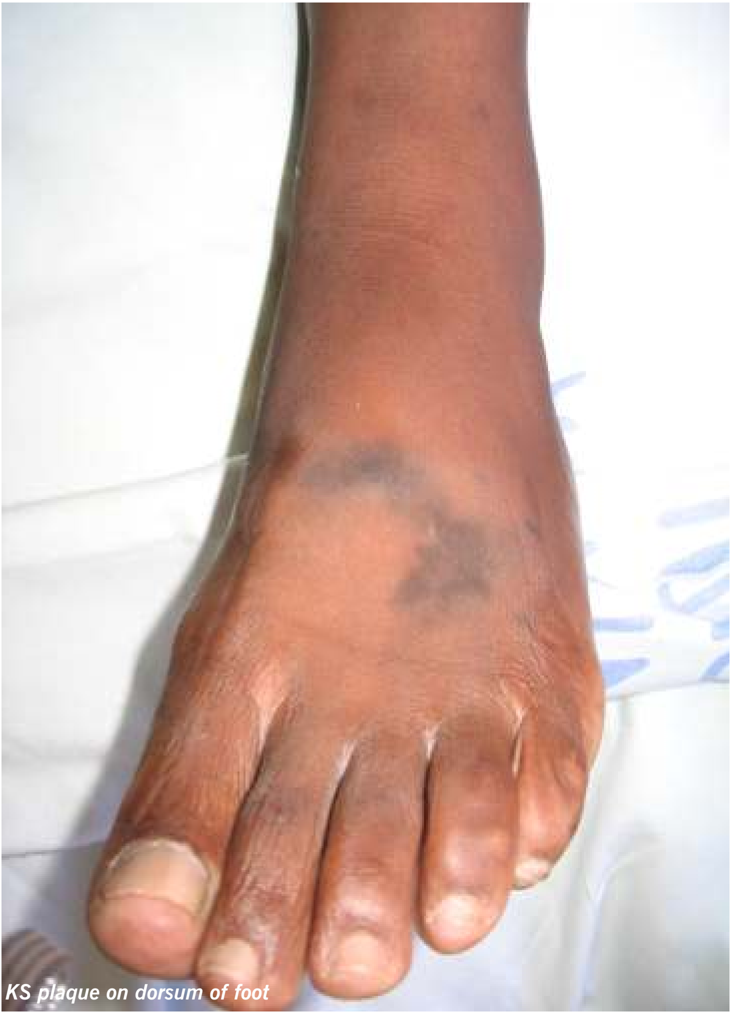

KS plaque on dorsum of foot

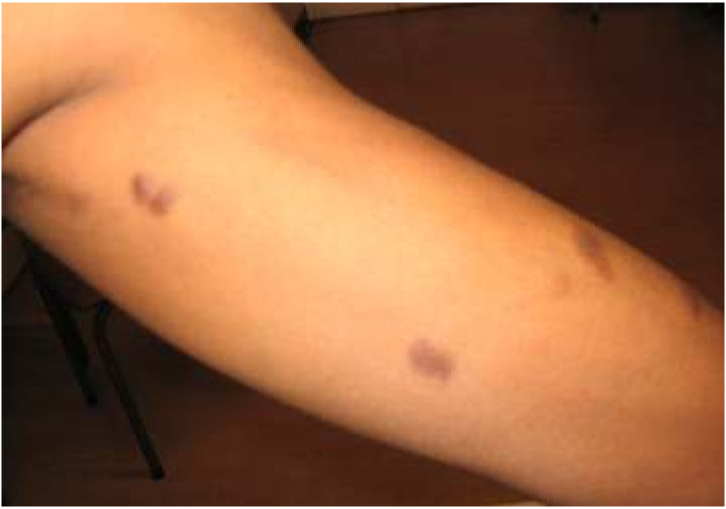

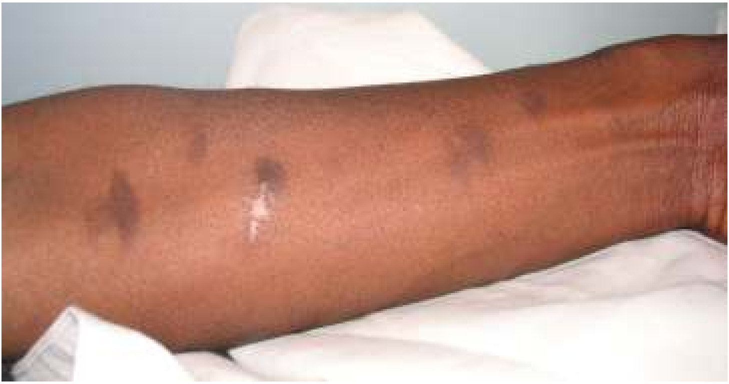

KS on the upper arm

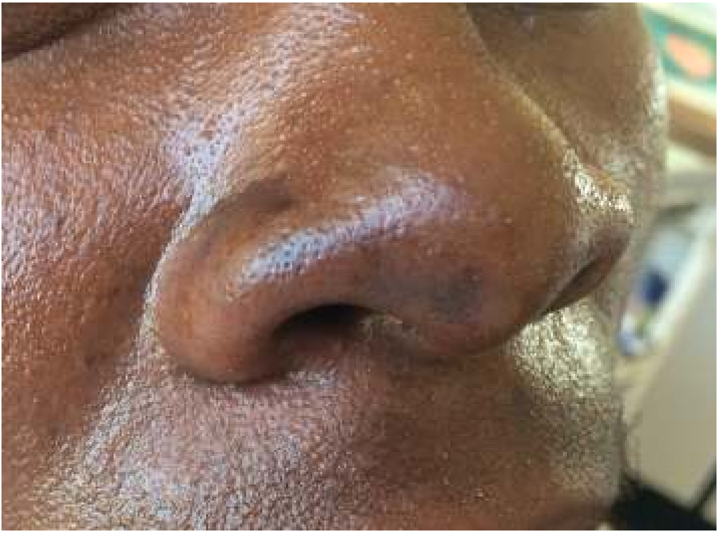

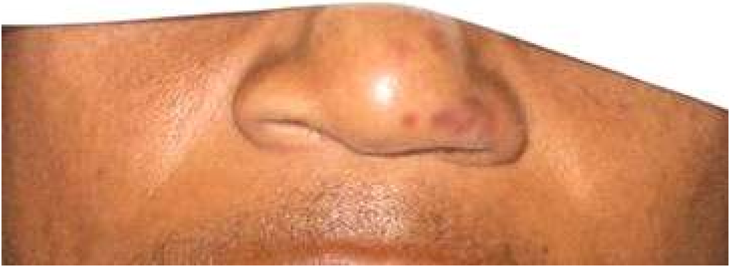

Early KS macules on the face

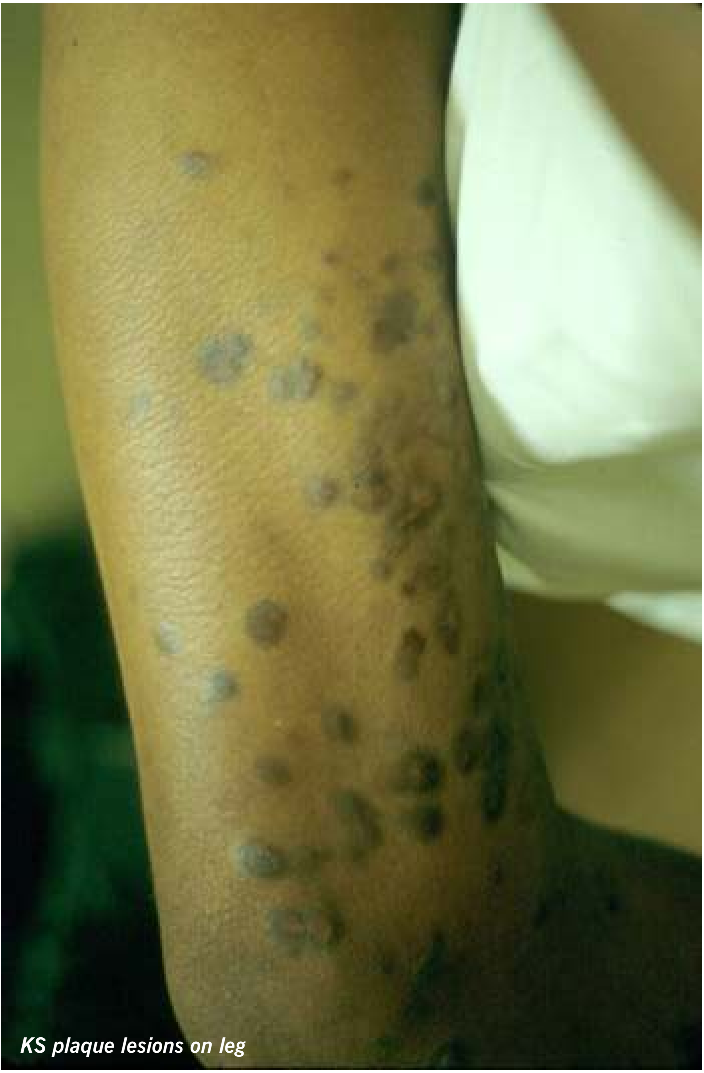

KS plaque lesions on leg

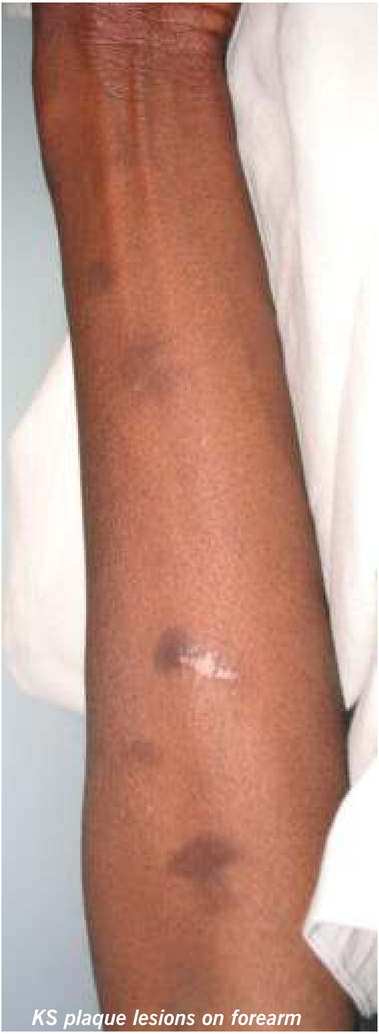

KS plaque lesions on forearm

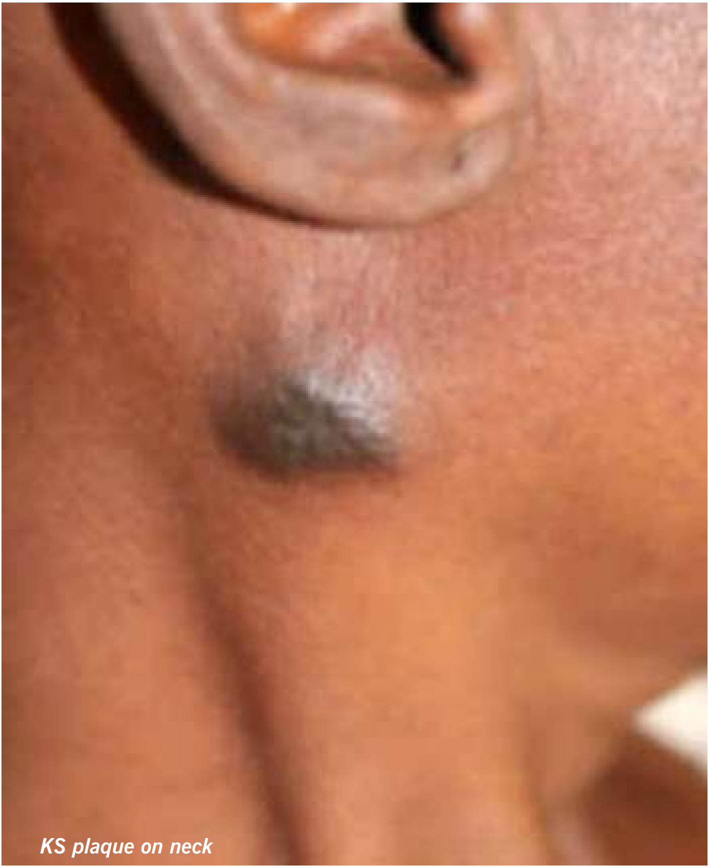

KS plaque on neck

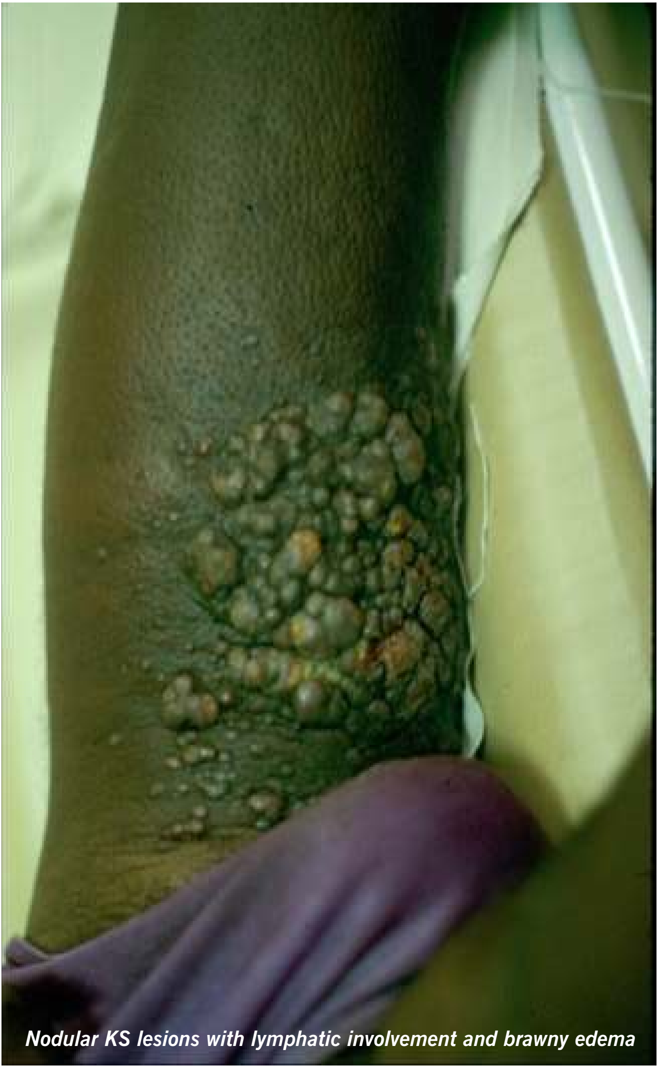

Nodular KS lesions with lymphatic involvement and brawny edema

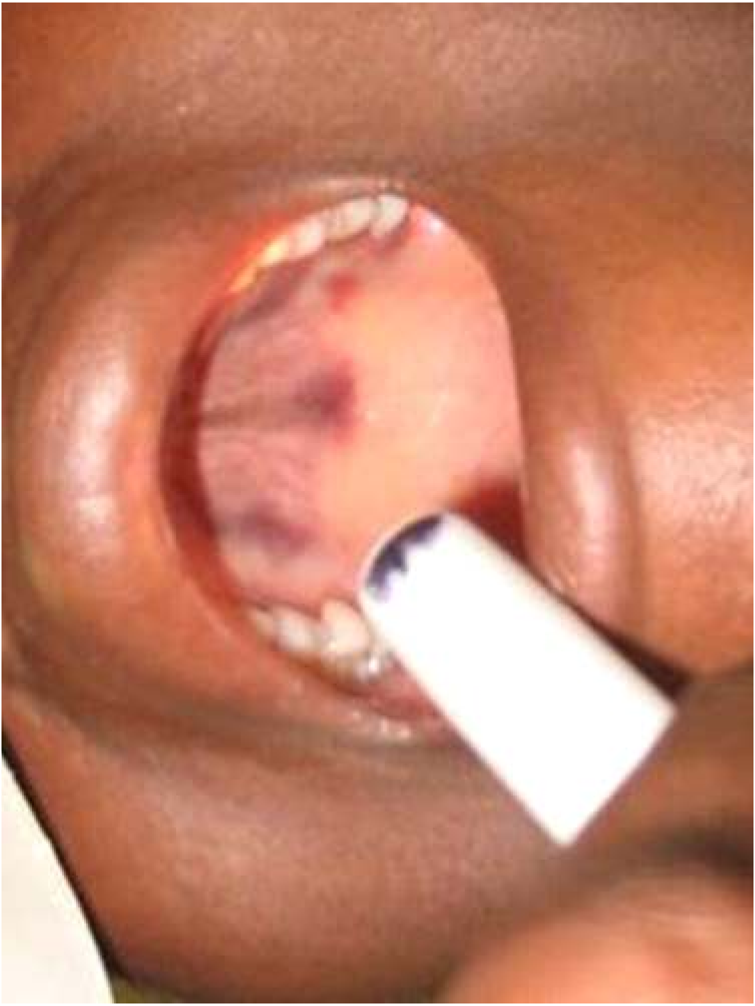

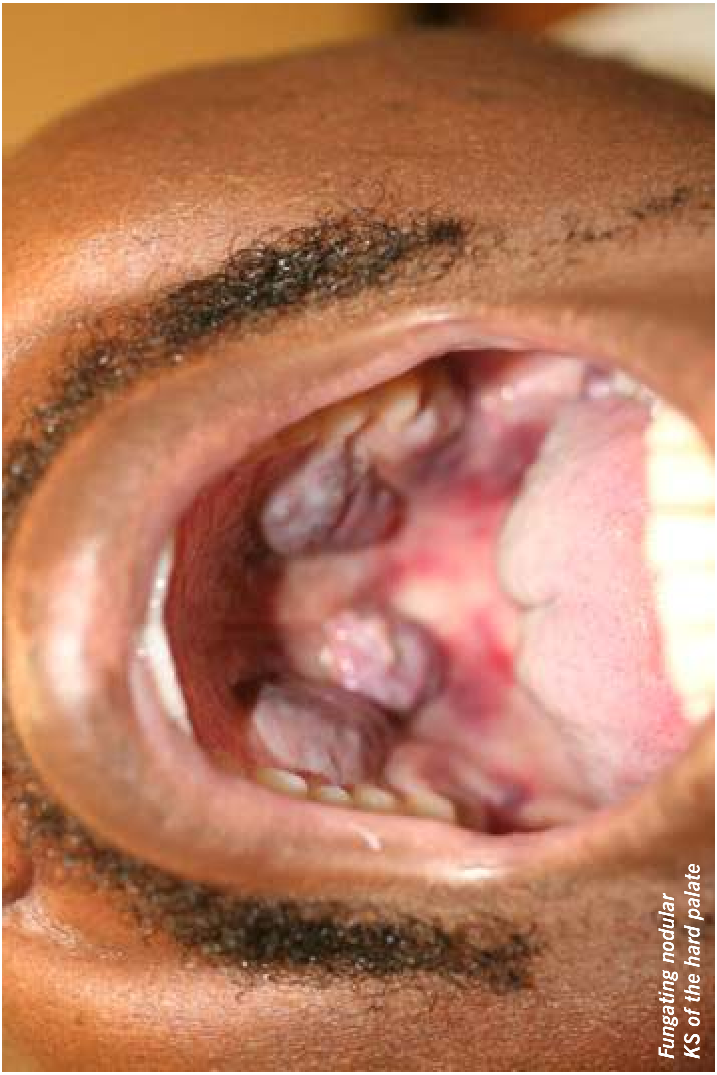

Fungating nodular
KS of the hard palate

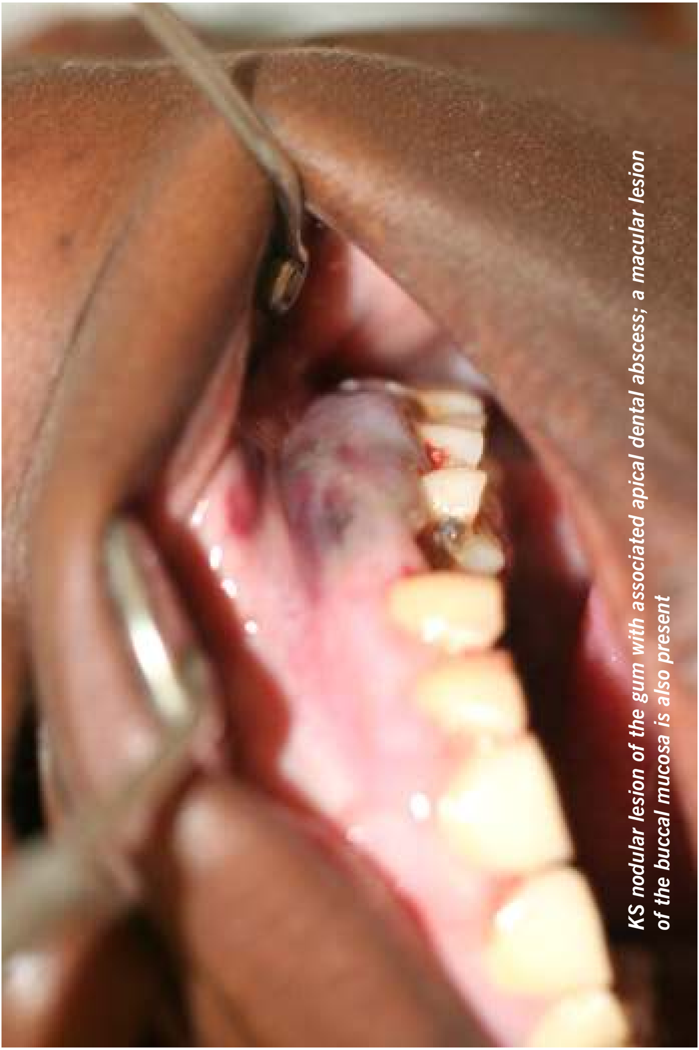

KS nodular lesion of the gum with associated apical dental abscess; a macular lesion of the buccal mucosa is also present

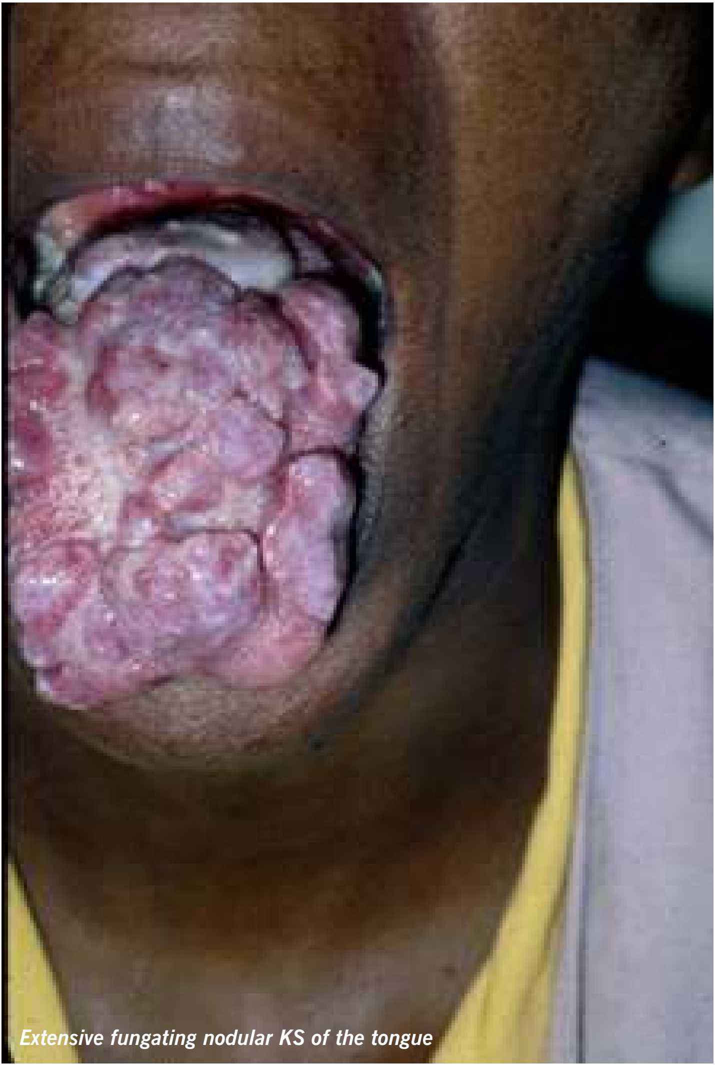

Extensive fungating nodular KS of the tongue

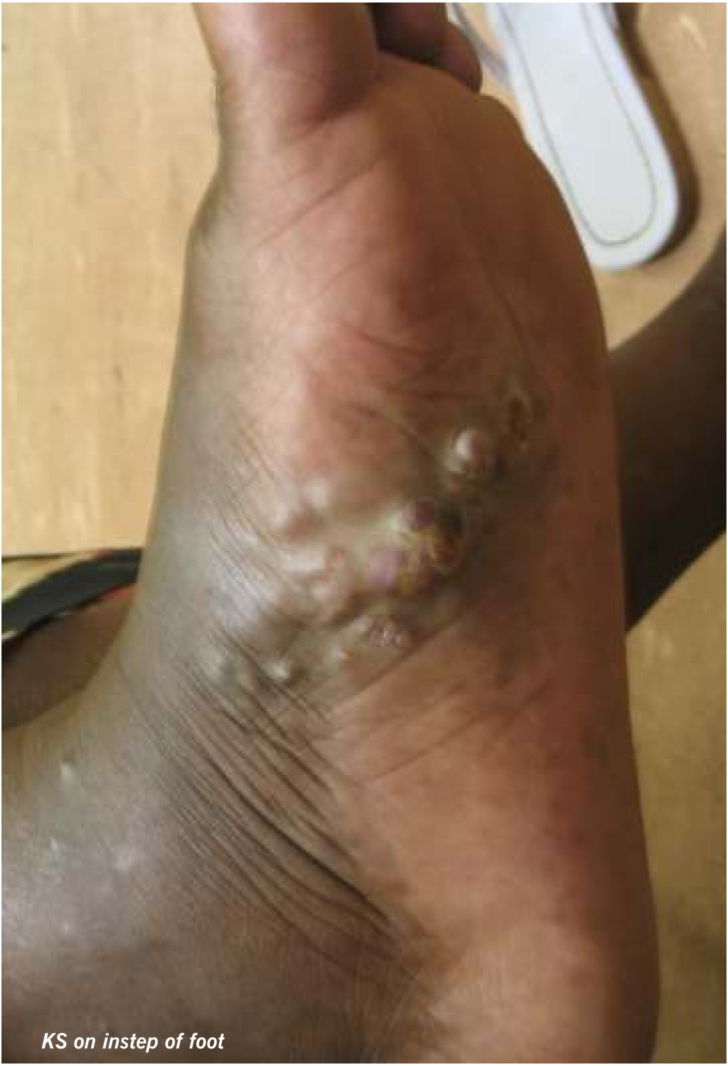

KS on instep of foot

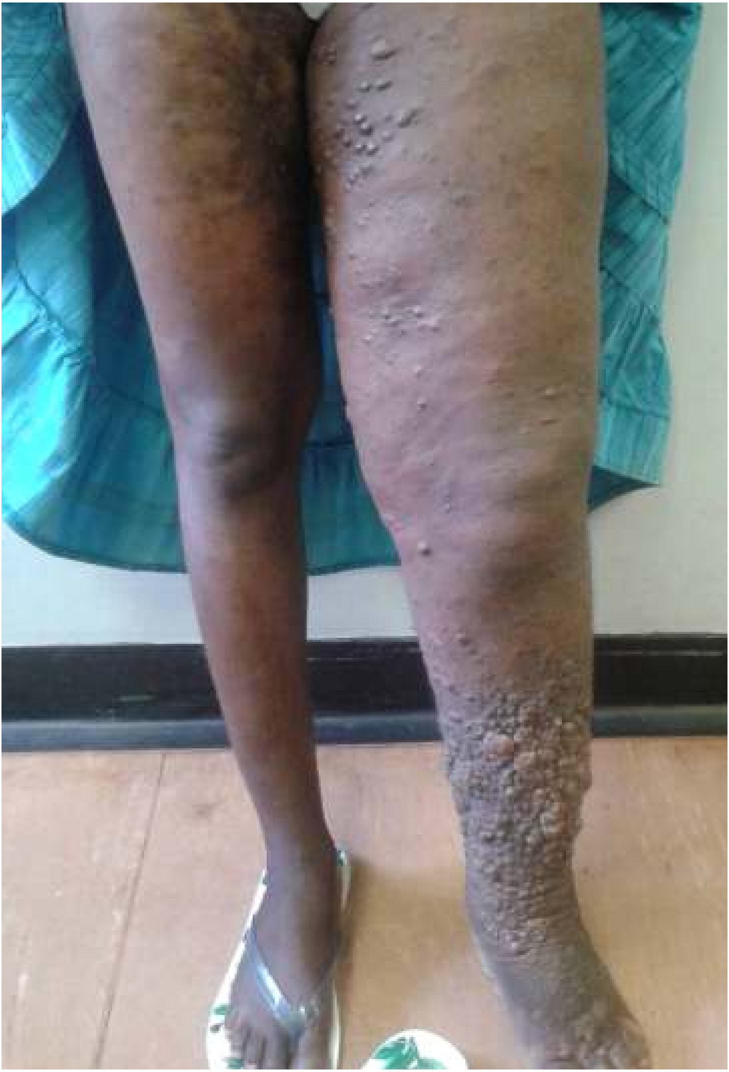

1.3 KS Staging

ACTG Staging Criteria: T (Tumour) I (Immune Status) S (Systemic symptoms)

Grade each of these categories (TIS) as '0' or '1', that is, good (0) or poor (1) prognosis

WHO categorises clinical KS as 'mild/moderate' or 'severe/symptomatic'.

In most cases, T0 usually refers to 'mild/moderate' KS and T1 to severe KS. There may be some overlap in patients with moderate disease, and clinical judgement is needed to define these categories further.

T0

- Confined to skin and/or lymph nodes and/or minimal oral disease.
- Minimal oral disease is defined as non-nodular KS confined to the palate, or oral disease that does not interfere with chewing or swallowing
- No significant oedema affecting function
- No symptomatic visceral disease
- Not life threatening or functionally disabling disease

T1

- Symptomatic tumor-associated edema
- Tumor ulceration
- Extensive oral KS (other than flat KS confined to the hard palate)
- Symptomatic visceral KS eg GIT, lung
- Life threatening or functionally disabling disease
- Progressive or persistent KS despite antiretrovirals

I0 CD4+ > 200 cells/ml

I1 CD4+ < 200 cells/ml

S0 no systemic symptoms

S1 presence of systemic symptoms – significant weight loss/sweats/fevers

Staging Examples

- 25 KS skin plaques and macules, KS in zoster scar, no leg edema, no oral KS, no constitutional symptoms.= T0S0
- 20 KS plaques with lymphoedema, plaque on tonsil, nodular lesions on chest X-ray, no respiratory symptoms, no evidence for lung infection, weight loss =T1S1
- 30 KS skin lesions, flat KS confined to the hard palate. Colonoscopy reveals several KS nodules in the sigmoid colon.=T1
- 40 KS skin lesions, flat KS confined to the hard palate, lymph node biopsy showing a focus of KS.=T0

1.4 KS treatment

KS is highly manageable. Often, early KS is manageable just by treating HIV infection with antiretrovirals. Late stage KS (T1) requires both antiretroviral treatment and chemotherapy. Radiation therapy may be used to improve local symptoms. Staging of KS is therefore important in the clinical assessment for development of an appropriate treatment plan, and to predict mortality. Since earlier stage disease (T0) responds better to

treatment, screening for KS and earlier detection of disease is an important component of HIV care. Bacterial, fungal and other infections, and immune reconstitution inflammatory syndrome (IRIS) are important complications after starting antiretroviral therapy.

2. Kaposi's Sarcoma Standardized Evaluation

HIV positive patients should be examined at least annually to detect evidence of KS.

Skin KS Disease

- Ask the patient if they have experienced any new or worrying skin lesions
- The patient should undress to allow complete skin examination
- Inspect the skin in all the following body areas: scalp, face, neck, back, chest, abdomen, buttocks, groin, arms, hands, legs and feet.
- The examiner should note the size and location of any hyperpigmented lesions and whether the lesions are flat (macules) or raised (papules, plaques or nodules). The number of suspicious lesions in each body area should be estimated.

The following is a guide to use to record severity of the skin

Number of lesions	Severity
· < 5	· scanty
· 5 < 20	· mild
· 20 < 50	· moderate
· > 50	· severe

- If lesions that are suspicious for KS are found, photograph the lesion for later consultation with a specialist if needed.

Oral KS Disease

- Ask the patient if they have noticed any troubling sores or masses in their mouth or on the tongue.
- *Lips:* Observe the lips, with the mouth both closed and opened. Note the color, texture, and any surface abnormalities of the upper and lower lip.
- *Lip (labial) Mucosa:* With the mouth partially open, examine the lower lip mucosa by pulling the it downward. Do the same for the examination of the upper lip mucosa by lifting the upper lip.
- *Buccal Mucosa and Vestibules:* With the mouth open wide, using a tongue depressor, examine first the right buccal mucosa (inside of cheek) extending from the corner of the lips and back to the anterior tonsillar pillar. Examine the left buccal mucosa in the same way.
- *Hard and Soft Palate:* With the mouth wide open and the patient's head tilted backwards, gently depress the base of the tongue with the mouth mirror or a spatula. First, inspect the hard palate located in the anterior part, and then the soft palate and uvula (ask the patient to say “ahhh” to better visualize the soft palate).
- *Tongue:* With the patient’s tongue at rest and mouth partially open, inspect the dorsum of the tongue for any swelling, ulceration, coating or variation in size, color, or texture. The patient should then put out the tongue to allow examination of the lateral borders. Then observe the under surface (ventral surface).
- *Floor of Mouth:* With the tongue still elevated, inspect the floor of the mouth.

- *Gums:* First, examine the buccal and labial aspects of the gums and alveolar ridge. Start with the right maxillary posterior gum and alveolar ridge and move around the arch to the left posterior gum. Continue with the left mandibular posterior gum and alveolar ridge and move around the arch to the right posterior gum. Second, examine the palatal and lingual aspects as has been done on the facial side, from right to left on the palatal (maxilla) and left to right on the lingual (mandible) side. Use the tongue depressor and focus the light Record the presence or absence of oral cavity KS lesions and their location and record whether lesions are raised or flat.

Internal Organs

- Ask about the presence of gastro-intestinal symptoms (nausea, vomiting, rectal bleeding, and/or abdominal pain) or respiratory symptoms (chronic cough, haemoptysis, dyspnea, chest pain).
- Record pulse and respiratory rate
- Observe whether breathing is labored or if splinting is present
- Carefully percuss the lungs noting dullness if present
- Auscultate the lungs noting any breath sound asymmetry and, if abnormal sounds, particularly wheezes are present
- Observe the size and contour of the abdomen
- Palpate the abdomen, note any masses, hepatomegaly, splenomegaly or ascites
- If abnormalities are detected on examination of the chest, obtain a chest x-ray if available

Lymphatics

- Patients should be asked about the presence of enlarged lymph nodes and swelling of the arms or legs, particularly if associated with pain
- Note whether there is any asymmetry in the size of the upper or lower extremities
- If edema is present note whether it is pitting or non-pitting and if there is any discoloration of the overlying skin
- Examine the neck, supraclavicular areas, axillae, epitrochlear spaces and inguinal and femoral areas for lymph nodes. If nodes are detected, note their location, size, size, contour and mobility

3. Photography of KS lesions

Photographs are often useful to assist in documentation of the diagnosis of KS and for clinical monitoring purposes. It is difficult to standardize these photographs. Patients should have photos of larger views of the back, chest, arms (front and back), legs (front and back), feet (including soles), whether involved with KS or not. In addition, photos should be taken of any other area with significant involvement at entry (e.g., the face). Appropriate measures must be taken to protect participant confidentiality and where possible, photographs of participants' faces should be avoided. In cases where a participant's face is photographed, no participant photos should be used in publication without patient permission and IRB approval; in addition removal of identifying characteristics, for example, the blacking out of a participant's eyes must be done

Photography Tips

1. Use a camera or phone camera (recommend 5 megapixels)
2. If possible, include the patient number in all of the photos.
3. Always try to take the photos in the same setting with respect to participant positioning, lighting, background, and camera setting.
4. Use auto-focus.
5. Use the “macro” mode for close-ups.
6. Use the flash mode if the lighting is poor, but avoid getting too close to the lesions since overexposure may wipe out the details.

See Appendix 2 for photographic tips.

4. Biopsy of cutaneous KS lesions

Materials

a) Scissors
b) Disposable Punch biopsy instrument (should be requested)
c) Cotton swab
d) 10% buffered Formalin in labeled container
e) Forceps

f) Sterile Fenestrated dressing towel
g) Sterile gloves
h) Sterile gauze
I) Bandages
j) Plaster
k) Betadine solution (or swabs)
l) Lidocaine local anesthetic
m) Needle/Syringe (25 gauge) for anesthetic

Procedure

1. The skin should be cleaned with betadine.
2. The sterile towel should be placed over the area.
3. Using a syringe, inject the lesion for biopsy and surrounding area with approximately 1mL of 1% lidocaine or 1% lidocaine with epinephrine, 1:100,000.
4. Allow the area to become numb (this takes only about 1-2 minutes).
5. Gently rotate the punch biopsy into the lesion up to the hilt where the metal punch meets the plastic handle of the instrument and pull out the punch.
6. Using forceps, grasp the punched lesion. You may need to detach the biopsy specimen from the base scissors.
7. The specimen should be placed in the appropriate container.
8. The sample should be placed in formalin and labeled using a fine marker.
9. Apply pressure to the wound with a gauze pad.
10. One may need to place asuture to aid hemostatis.
11. Dress the wound with and plaster bandage.
12. Transport biopsy specimen to lab within 5 days.

5. KS Management algorithm

The SIKO strategy for KS management has two objectives:

1) Diagnose KS at the earliest stage possible to help ensure the better responses to treatment.

2) At the time of diagnosis, perform comprehensive assessments of KS disease including clinical tumor staging and biopsy to confirm the diagnosis.

3) Institute appropriate KS treatment in the primary care setting with the assistance of SIKO consultants.

Algorithm-based Management Strategy

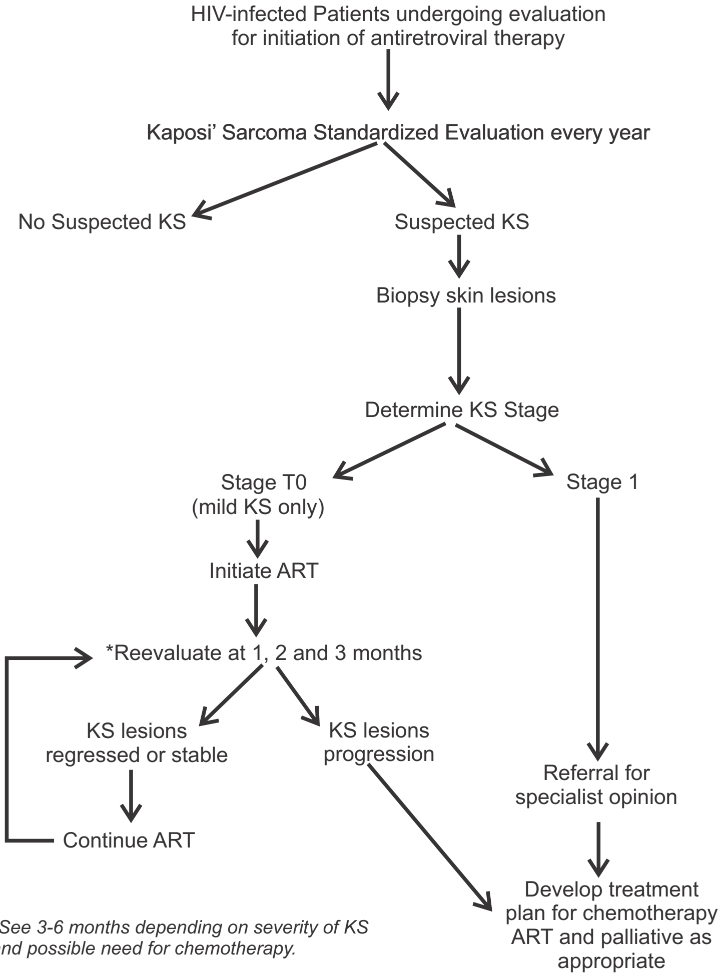

**See 3-6 months depending on severity of KS and possible need for chemotherapy.*

NB. Where possible TB should be excluded before starting chemotherapy

Treatment of KS

- No known curative therapy for KS
- Goals of treatment:
  - Palliation of symptoms
  - Shrinkage of tumour to alleviate oedema and to improve organ function
  - Prevent disease progression
  - Relieve psychological stress

Treatment Options

- HAART
- Chemotherapy
- Other treatment
- Radiotherapy

1. **Antiretroviral therapy (ART)** – all patients with AIDS-KS require ART. The timing of the ART is important so as to avoid KS IRIS.

2. **Chemotherapy** – should be given in consultation with the specialist KS Clinic, and usually at a central hospital
   a. bleomycin/vincristine/doxorubicin/daunorubicin – combination of 2 of these
   b. taxanes (paclitaxel) – second line chemotherapy

Where possible TB should be excluded before starting chemotherapy

3. **Additional/other treatment includes several newer agents** – the evidence for their use is variable. These include angiogenesis inhibitors eg. Thalidomide, Imatinib which is orally active tyrosine kinase inhibitor, Vitamin D and analogues, interferon- which is a biological response modifier, anti KSHV therapy

4. **Radiotherapy**
    a. Local – bleeding, ulcerating fungating lesions, pain
    b. Hemi-body irradiation

5. **Local therapy** – rarely used for minimal disease only, confined to a small area, cosmetically or functionally troublesome. Modalities include topical retinoids, intra-lesional chemotherapy, external beam irradiation, laser therapy, cryotherapy, excisional surgery

NB PLAN THE TREATMENT – TIMING IS IMPORTANT

WHO guidelines – KS is in Stage 4

KS IRIS does occur and should be avoided if possible

ALWAYS assess first for need for chemotherapy to reduce tumour bulk and stabilize the patient BEFORE using ART

Manage the patient in consultation with the KS referral clinic or a specialist HIV/Oncology team

KS IRIS

- Worsening of existing KS with ART
- Development of new lesions with ART
- Occurs in the situation of a rapid decline in HIV VL and increase in CD4 of 50 cells over 12 weeks
- Close monitoring required
- Pulmonary/pleural involvement may be fatal
- HAART is continued, but chemo is needed

PROGNOSIS OF KS

- Depends on the HIV disease and the response to ART
- Prognosis of KS
    - Often a late diagnosis
    - Often a heavy tumour burden
    - KS IRIS may occur

- availability of chemotherapy and other treatment
- palliative management should start at diagnosis, especially control of pain and breathlessness

6. Obtaining KS and Palliative Specialty Consultation

Referral to Kaposi Clinic, Parirenyatwa Hospital, every Thursday, appointment preferred but not essential.
Biopsy result is not essential before referral. If biopsy has been done the result can usually be traced.
Chest Xray and FBC are very helpful.

PGH Telephone: +263 4 701555/9
Prof. MZ Borok +263 712400713 (mborok@mweb.co.zw)
Dr. P Chidawanyika +263 772844 565
Dr. G Munyaradzi +263 737 403421

Appendix 1: WHO Guidelines (2014)

TOPIC	RECOMMENDATIONS
**Kaposi sarcoma**	**Mild/moderate disease:** In HIV-infected adults, adolescents and children diagnosed with mild/moderate Kaposi sarcoma, immediate ART initiation is recommended. *(Strong recommendation, low quality evidence)*
	**Severe/symptomatic disease:** In HIV-infected adults, adolescents and children diagnosed with severe symptomatic Kaposi sarcoma, immediate ART initiation in combination with systemic chemotherapy is recommended. *(Strong recommendation, low quality evidence)*
	Recommended chemotherapy regimens in adults, adolescents and children may include vincristine with bleomycin and doxorubicin (ABV), bleomycin with vincristine (BV), and when available or feasible, liposomal anthracyclines (doxorubicin or daunorubicin), paclitaxel or oral etoposide at sites with the infrastructure, staff and resources to administer chemotherapy drugs and provide appropriate monitoring and supportive care. *(Conditional recommendation, low quality evidence)*

Appendix 2: Extra photography tips

1. For very close shots, oblique views may be preferred.

2. Eliminate all distractions from the background. Try to take all photographs with a plain blue or green background.

3. Make it a point to take at least 2 shots from each point of focus. Minimal blurring may not be obvious on the LCD screen and may be noticeable only after the image is viewed on the monitor. It is always better to have an extra copy from every focus point so that the best image can be selected.

4. Always try to capture distinctive elements like typical representative lesions, particular configurations, or distribution patterns.

5. For generalized lesions take shots from at least three ranges:
   a) A complete vertical view of the participant showing the extent and distribution of the rash;
   b) A medium distance shot showing the arrangement and configuration of the rash;
   c) A close-up view highlighting a representative lesion.

6. For localized lesions take shots from at least two points:
   a) A medium view showing the rash /lesion with respect to location and configuration
   b) Always include a recognizable body landmark so that the location is obvious. For example, for lesions on the abdomen include the umbilicus in the medium distance shot)

c) A close-up view of the representative lesion

7. For isolated lesions it is also advisable to include a discernible landmark in one of the shots. For the close-up shots use a measuring tape/ruler in the frame to demonstrate the size of the lesion. It would be advisable to take the close-up shots from more than one angle and include oblique shots. Shots with and without flash may be taken and the best shot selected for storage.

Saving, storing, and uploading files

1. SAVE as a JPG file. The major advantage of the JPG format is that the image size can be compressed considerably without significant visible loss of resolution. The back-up copies can also be saved in the compressed JPG format so that the space taken up can be minimized.
2. Delete images that are blurred.
3. Make it a point to catalogue all saved images (or containing folders) tagging them with the participant's name, hospital number, date and even the provisional diagnosis, if possible. Meticulous cataloging may seem cumbersome at the beginning but makes future retrieval of images easier.
